# The Effect of Autologous Dendritic Cell Immunotherapy on Kidney Function and Endothelial Dysfunction of Patients with Diabetic Kidney Disease (DKD): An Open Label Clinical Trial

**DOI:** 10.3390/cimb47010031

**Published:** 2025-01-06

**Authors:** Martina Lily Yana, Enda Cindylosa Sitepu, Jonny, Linda Chiuman, I Nyoman Ehrich Lister, Terawan Agus Putranto

**Affiliations:** 1 Indonesia Army Cellcure Center, Gatot Soebroto Central Army Hospital, Jakarta 10410, Indonesia; martinalily@yahoo.com (M.L.Y.); endacsitepu@gmail.com (E.C.S.); jonny@unprimdn.ac.id (J.); 2 Department of Clinical Pathology, Gatot Soebroto Central Army Hospital, Jakarta 10410, Indonesia; 3 Faculty of Medicine, Dentistry, and Health Science, Universitas Prima Indonesia, Medan 20118, Indonesia; lindachiuman@unprimdn.ac.id (L.C.); nyoman@unprimdn.ac.id (I.N.E.L.); 4 Faculty of Medicine, Universitas Pembangunan Nasional “Veteran” Jakarta, Jakarta 12450, Indonesia; 5 Nephrology Division, Department of Internal Medicine, Gatot Soebroto Central Army Hospital, Jakarta 10410, Indonesia; 6 Faculty of Military Medicine, Indonesia Defence University, Bogor 16810, Indonesia

**Keywords:** dendritic cell, immunotherapy, cell transfer, diabetic kidney disease, endothelial dysfunction

## Abstract

This study aimed to evaluate the effects of autologous dendritic cell (DC) immunotherapy on clinical outcomes (glomerular filtration rate/GFR and urine creatinine albumin ratio/UACR) and endothelial dysfunction (ICAM, VCAM, VEGF) in patients with diabetic kidney disease (DKD). Endothelial dysfunction induced by inflammation is one of the key factors in the pathogenesis of DKD. In this one-group pretest–posttest quasi-experimental study, 69 subjects with DKD were administered a single dose of autologous DC immunotherapy ex vivo. UACR was measured at baseline and at weeks 1, 2, 3, and 4, while ICAM, VCAM, VEGF, and GFR were measured at baseline and at week 4 post-immunotherapy. The results showed a significant reduction in median UACR from 250 (IQR 71–668) mg/g at baseline to 164 (IQR 49–576) mg/g at week 4 (*p* < 0.05). GFR did not show any significant changes after immunotherapy. HbA1c (B = −33.270, *p* = 0.021) and baseline UACR (B = −0.185, *p* < 0.001) were identified as significant predictors of UACR change. Although there were no significant changes in ICAM, VCAM, and VEGF, subgroup analysis revealed a decrease in VCAM in macroalbuminuria patients and an increase in those with good glycemic control, suggesting differing endothelial responses. In conclusion, autologous DC immunotherapy effectively reduced UACR in DKD patients, and significant VCAM changes were found in macroalbuminuria and good glycemic control subjects. Further research is needed to understand the mechanisms behind UACR reduction and the long-term impact of this therapy.

## 1. Introduction

DKD is a clinical syndrome characterized by pathological features such as persistent albuminuria, progressive decline in renal function, and histological changes in the kidneys [1]. Structural and functional changes include glomerular mesangial expansion, basement membrane thickening, podocyte loss, nodular glomerulosclerosis, and endothelial cell damage. In the early stages, tubular hypertrophy occurs, which later progresses to interstitial fibrosis with tubular atrophy [2].

One of the key pathological features of DKD is endothelial damage in the glomerular basement membrane, which leads to impaired kidney filtration, resulting in albuminuria [3]. Endothelial dysfunction in DKD is closely related to persistent hyperglycemia, a hallmark of diabetes. High blood glucose levels lead to the formation of advanced glycation end products (AGEs) which, through interaction with the AGE receptor (RAGE), activate various inflammatory pathways [4,5,6,7]. This process mediates the development of a low-grade chronic inflammatory condition in diabetes [8]. This complex interaction involves multiple biochemical and molecular pathways, with chronic inflammation acting as a major driver in disrupting endothelial homeostasis and contributing to the progression of DKD [9]. Chronic inflammation triggers the formation of reactive oxygen species (ROS), which cause oxidative stress. Oxidative stress disrupts the synthesis and availability of nitric oxide, a crucial molecule for endothelial function responsible for vasodilation, maintaining blood flow, and inhibiting platelet aggregation [10].

Another key aspect of inflammation-induced endothelial dysfunction in DKD is activating the nuclear factor kappa-light-chain-enhancer (NF-κB) pathway. This pathway is central to the inflammatory response, and its activation in endothelial cells increases the expression of various adhesion molecules such as ICAM (intercellular adhesion molecule) and VCAM (vascular cell adhesion molecule). The elevated expression of these molecules facilitates leukocyte adhesion and migration to the endothelium, further exacerbating endothelial damage [11]. VEGF (vascular endothelial growth factor) is upregulated as a compensatory response to endothelial damage. VEGF plays a crucial role in angiogenesis, endothelial cell proliferation and survival, and tissue regeneration in the kidneys. Therefore, in the early stages of kidney damage, VEGF levels increase. However, in severe kidney damage, VEGF levels decrease due to extensive damage where kidney cells can no longer express VEGF [12].

The use of angiotensin-converting enzyme (ACE) inhibitors or angiotensin receptor blockers (ARBs) has been proven to reduce proteinuria and slow the decline in kidney function. Recent studies have highlighted the impact of new pharmacological agents, such as sodium-glucose cotransporter 2 (SGLT2) inhibitors, on the management of diabetic kidney disease (DKD). Empagliflozin, an SGLT2 inhibitor, has demonstrated significant renal and cardiovascular benefits in patients with type 2 diabetes and pre-existing kidney disease [13]. However, the efficacy of these drugs is generally unsatisfactory. Furthermore, ACE inhibitors, can cause various side effects such as hyperkalemia, angioedema, and decreased renal function that can be life threatening [14,15,16]. Therefore, it is essential to explore alternative approaches that can enhance the effectiveness of current treatments while minimizing their side effects.

One promising avenue lies in targeting the immune system, which plays a central role in the progression of DKD and other chronic inflammatory conditions. In this context, dendritic cells (DCs) have garnered significant attention due to their pivotal role in bridging innate and adaptive immunity. DCs are unique in their ability to process and present antigens to other immune cells, which is vital in regulating immune responses [17]. In inflammatory conditions, DCs play a dual role. They can trigger immune responses to fight infection and cause inflammation, but they also have the ability to control excessive inflammatory responses. This is achieved by inducing regulatory T cells (Tregs) and producing anti-inflammatory cytokines. Thus, DCs are crucial in maintaining immune balance and preventing autoimmune diseases as well as excessive inflammatory responses that can damage body tissues [18].

DCs can be generated outside the body by isolating peripheral blood monocytes and culturing them in differentiation media containing granulocyte–macrophage-stimulating factor (GM-CSF) and interleukin-4 (IL-4) [19]. These ex vivo-generated DCs are then transferred back to the patient’s body, where they efficiently migrate to the lymph nodes and perform their function in vivo. Autologous DC transfer can potentially improve and prevent disease progression in DKD patients [20]. However, to date, no studies have investigated the effect of autologous DC administration on clinical outcomes in DKD. Given the critical role of endothelial dysfunction in the pathogenesis of DKD and the role of DCs in maintaining endothelial function, further studies are needed to assess the effect of DC immunotherapy on endothelial dysfunction. Therefore, this study investigated the effect of DC immunotherapy on endothelial dysfunction in DKD patients.

## 2. Materials and Methods

### 2.1. Study Design

This study is an open-label clinical trial without randomization and without the use of blinding. The study design is quasi-experimental with a pretest and posttest approach. Sham–control group, blinding, and placebo are not utilized due to ethical considerations. The research procedures were developed in accordance with applicable guidelines and regulations, ensuring subject protection and ethical compliance. This study has received approval from the Ethics Committee of Gatot Soebroto Army Central Hospital (RSPAD GS) with Ethical Clearance Number 108/VIII/KEPK/2024. All subjects provided written informed consent before participating.

### 2.2. Study Subjects

The research subjects were DKD patients receiving outpatient care at the internal medicine clinic of RSPAD GS from April to May 2024. Sampling was conducted using a nonprobability sampling method, specifically consecutive sampling (quota sampling). Based on the calculations, a minimum of 67 subjects were required to detect significant changes in the urine albumin–creatinine ratio (UACR). The subjects included in this study (inclusion criteria) were diabetic patients fulfilling criteria from the Indonesian Endocrinology Association (PERKENI) 2021 guidelines, aged over 18 years, with an eGFR of ≥30 mL/min/1.73 m^2^ and a urinary albumin–creatinine ratio (UACR) of ≥30 mg/g. However, the study excluded patients known to be receiving immunosuppressive treatment, anticancer therapy (except hormonal therapy), or antithrombotic treatment other than low-dose aspirin. Patients with other kidney diseases, other forms of diabetes, cancer, immunodeficiency diseases, or those receiving oxygen supplementation therapy were also excluded from the study.

The sample size was determined using sample size formula for comparing means in numerical data for a single population.

(1) $$n={\left(\frac{\left[Z_{\alpha }+Z_{\beta }\right]S}{x_{1}-x_{2}}\right)}^{2}\;n:Sample\;Size\;S:Standard\;Deviation\;x1-x2:Effect\;Size\;Z\alpha :Z\text{-}score\;with\;alpha\;0.05=1.64\;Z:Z\text{-}score\;with\;beta\;(power)\;0.8=0.84$$

Based on a previous study by Kashiwagi et al., the standard deviation (σ) of UACR in patients with T2DM is 497.8 mg/g [21]. Using a confidence level of α = 0.05, a power of 0.8, and an expected change in UACR of 150 mg/g, the minimum sample size for this study was 68 subjects. Post hoc statistical power calculation for the multivariate model is described in Section 2.4.

### 2.3. Study Procedure

The study lasted for five weeks and began with a screening phase, where the subjects’ conditions were evaluated to ensure they met the inclusion criteria. After passing the screening, subjects underwent laboratory tests to collect baseline data, including clinical parameters, and a blood draw of 40 cc to generate dendritic cells (DCs). DC and antigens were prepared as described in previous studies [22,23]. To summarize, the procedure was as follows: The peripheral blood was processed to isolate peripheral blood mononuclear cells (PBMCs) based on density gradient medium using Lymphoprep^TM^ (Stemcell^TM^ Technologies Inc., Vancouver, BC, Canada), which were then incubated with MoDC differentiation media supplemented with granulocyte macrophage colony stimulating factor and interleukin-4 (Aivita Biomedical, Irvine, CA, USA) for five days in 37 °C with 5% CO_2_. Subsequently, maturation was initiated by incubating the DCs with antigens for two days (Aivita Biomedical, Irvine, CA, USA). The cell products were injected subcutaneously by a trained physician. Subjects were closely monitored for 30 min post administration to observe for any signs of allergic reactions. Any adverse events were documented for up to seven days following the injections. Moreover, all subjects continued their routine therapy without any modifications throughout the study period.

Following the DC administration, subjects’ conditions were monitored through weekly evaluations of the urine albumin–creatinine ratio (UACR) over a four-week period. Further laboratory evaluations were conducted in the fourth week after injection, including measuring endothelial dysfunction biomarkers (ICAM, VCAM, and VEGF) and Creatinine to monitor the estimated glomerular filtration rate (eGFR). The Urine Albumin Creatinine Ratio was assessed using the turbidimetric method, while ICAM, VCAM, and VEGF were measured from serum using a sandwich-ELISA kit (Reed Biotech Ltd., Wuhan, China). All stages of this study were carried out in accordance with the protocol approved by the Health Research Ethics Committee of RSPAD GS (No. 108/VIII/KEPK/2024), ensuring compliance with ethical standards and subject safety.

### 2.4. Statistics

The median UACR at weeks 1, 2, 3, and 4 post intervention was compared to baseline. The median eGFR at week 4 post intervention was also compared to baseline. Endothelial dysfunction biomarkers at week 4 post intervention were compared to baseline. Normality testing of the data was conducted using the Shapiro–Wilk test. Hypothesis testing for normally distributed data was performed using the paired t-test, while non-normally distributed data were analyzed using the Wilcoxon sign rank test. Spearman correlation analysis was then conducted for each parameter at baseline and week 4 post intervention. A multivariate analysis was performed to account for the effects of confounding factors, which in this study included age, baseline UACR, HbA1c, eGFR, and concomitant drug use. By the end of the study, the total sample size was 69 subjects. Post hoc power analysis for the multivariate model was conducted using G-power [24]. With a medium effect size of (0.15), a significance level of α = 0.05, 5 predictors, and a sample size of 69, the resulting statistical power (1-β) was 0.65. However, this is below the generally accepted threshold of 0.80 for adequate power. A *p*-value < 0.05 was considered statistically significant. Statistical analysis was performed using SPSS version 13 (IBM, Armonk, NY, USA), and data visualization was conducted using Graphpad Prism 8 (Graphpad Software, LLC, San Diego, CA, USA).

## 3. Results

### 3.1. Subject Characteristics

A total of 69 subjects completed the study and were analyzed. The average age of the subjects was 62 years, with an age range of 39 to 83 years. The gender ratio of the subjects (male:female) was 1:1.3. The majority of subjects were of Javanese ethnicity (42%), followed by Chinese (13%), Betawi (12%), Batak (12%), Sundanese (7%), Minangkabau (6%), and others (9%). A total of 94.2% of the subjects had hypertension, with most receiving ACE inhibitor therapy (63.8%). The majority of subjects were also on insulin therapy (63.8%). Based on body mass index (BMI), most of the subjects were categorized as overweight (50.7%), with median triglyceride levels of [145 (100–187) mg/dL], LDL [115 (93–158) mg/dL], and HDL [47 (41–53) mg/dL]. A total of 52.2% of the subjects had a UACR level < 300 mg/g (microalbuminuria), while 47.8% had a UACR level > 300 mg/g (macroalbuminuria). Based on eGFR criteria, most subjects had an eGFR of 30–44 mL/min/1.73 m^2^ (33.4%) and 60–89 mL/min/1.73 m^2^ (30.4%). A complete description of subject characteristics can be seen in Supplementary Table S1.

No serious adverse events were observed in this study. Adverse events were limited to local reactions such as pain and swelling at the injection site.

### 3.2. Clinical Outcome

**Figure 1:**
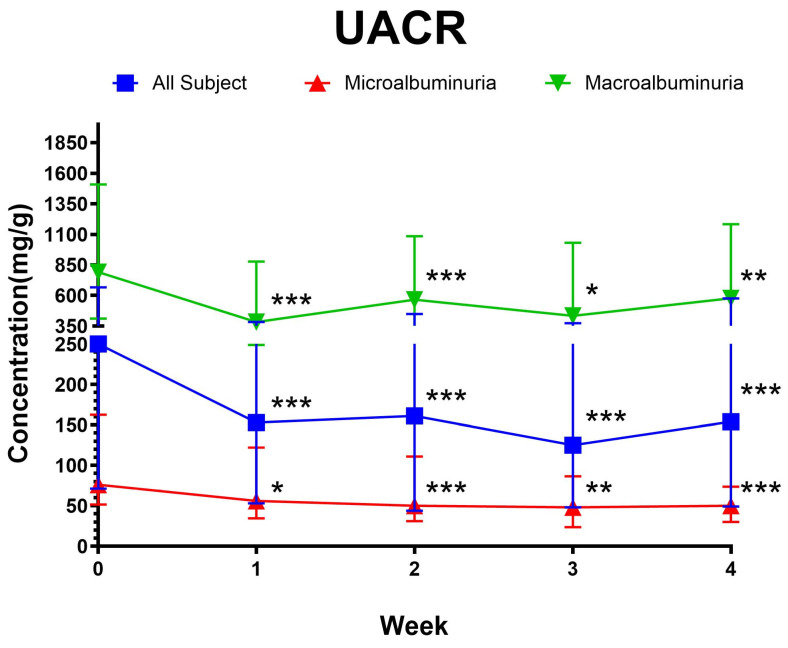
Weekly comparison of urine albumine–creatinine ratio (UACR). * *p* < 0.05; ** *p* < 0.01; *** *p* < 0.001; *p*-value was calculated in comparison to baseline (week 0) using the Wilcoxon sign rank test. Data were presented asa median ± interquartile range (IQR). All subjects *n* = 69, microalbuminuria *n* = 36, macroalbuminuria *n* = 36.

The change in median UACR (mg/g) in patients with diabetic kidney disease (DKD) following autologous dendritic cell immunotherapy is described in Figure 1. At baseline, the median UACR was 250 mg/g (IQR 71–668 mg/g). After therapy, a significant reduction compared to baseline UACR was observed at week one (153 mg/g; IQR 53–383 mg/g), week two (161 mg/g; IQR 44–448 mg/g), week three (125 mg/g; IQR 48–373 mg/g), and week four (164 mg/g; IQR 49–576 mg/g) compared to baseline, with all *p*-values < 0.05 (Wilcoxon sign rank test). Each week comparison shows that the reduction is sustained up to four weeks without any significant fluctuation (week 1 vs. week: *p* = 0.827; week 2 vs. week 3: *p* = 0.0263; week 3 vs. week 4: *p* = 0.623). This significant decrease in UACR indicates an improvement in kidney function related to albuminuria following immunotherapy.

Subjects were categorized into two subgroups based on their baseline albuminuria levels: microalbuminuria (UACR 30–300 mg/g) and macroalbuminuria (UACR > 300 mg/g). Participants in the microalbuminuria subgroup showed a significant reduction in UACR levels from baseline (76 mg/g; IQR 51.50–162.50 mg/g) to week one (56 mg/g; IQR 34.50–122.00 mg/g), week two (50 mg/g; IQR 31.00–111.00 mg/g), week three (48 mg/g; IQR 23.50–86.50 mg/g), and week four (50 mg/g; IQR 30.00–73.50 mg/g), with all reductions achieving statistical significance (*p* < 0.05, Wilcoxon signed rank test). Similarly, participants in the macroalbuminuria subgroup exhibited a significant reduction in UACR levels from baseline (792 mg/g; IQR 412.00–1508.00 mg/g) to week one (383 mg/g; IQR 249.00–878.00 mg/g), week two (566 mg/g; IQR 281.00–1086.00 mg/g), week three (434 mg/g; IQR 275.00–1031.00 mg/g), and week four (577 mg/g; IQR 296.00–1183.00 mg/g), all with *p* < 0.05 (Wilcoxon signed rank test). Weekly comparisons indicated that the reductions in UACR levels were sustained over the four-week period without significant fluctuations (*p* > 0.05). Additionally, the percentage reduction in UACR from baseline to week four was calculated, showing an overall reduction of 25.41% ± 36.40% (mean ± standard deviation) across all subjects. In the microalbuminuria subgroup, UACR decreased by 32.12% ± 39.05%, while the macroalbuminuria subgroup demonstrated a reduction of 18.09% ± 32.85%. However, the difference in percentage reduction between the two subgroups was not statistically significant (*p* = 0.113, independent *t*-test).

Table 1 presents the results of the median creatinine levels and estimated glomerular filtration rate (eGFR) analysis before and after autologous dendritic cell immunotherapy in patients with diabetic kidney disease (DKD). No significant changes to either creatinine or eGFR were detected (*p* > 0.05). These results suggest that immunotherapy did not lead to a noticeable deterioration nor improve the kidney function in terms of creatinine levels or eGFR following therapy.

### 3.3. Predictors of UACR Change

**Figure 2:**
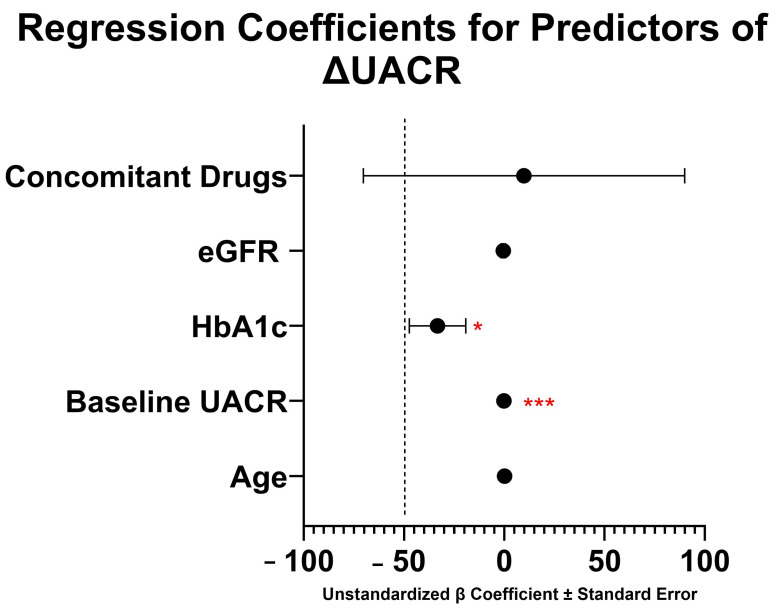
Regression coefficients with standard errors for predictors of ΔUACR. ΔUACR was calculated from the difference between UACR at week four and baseline. Independent variables significantly predict ΔUACR [F(5, 63) = 12.97; *p* < 0.001]. This model explains 50.7% of variance in ΔUACR (R^2^ = 0.507). Constant (β_0_) in this model is 292.27 ± 255.90. * *p* < 0.05, *** *p* < 0.001, eGFR: estimated glomerular filtration rate, HbA1c: A1c hemoglobin, UACR: urine albumin–creatinine ratio.

Multivariate analysis using multiple linear regression was conducted for potential confounding factors (Figure 2). Concomitant drug use was defined as medication that affect UACR such as RAAS inhibitors, diuretics, and SGLT-2. ΔUACR was calculated as the difference between UACR at week 4 and UACR at baseline. The results of the multiple linear regression analysis, with ΔUACR as the dependent variable, are presented in Figure 2. The model revealed that baseline UACR (B = −0.185, *p* < 0.001) and HbA1c (B = −33.270, *p* = 0.021) were significant predictors of ΔUACR. Baseline UACR demonstrated a strong negative association with changes in UACR, indicating that higher baseline UACR values were associated with a greater reduction in UACR. Similarly, HbA1c showed a significant negative relationship, suggesting that poorer glycemic control was associated with a smaller improvement in UACR. In contrast, the variables concomitant drugs (B = 9.839, *p* = 0.903), age (B = 0.133, *p* = 0.966), and eGFR (B = −0.487, *p* = 0.661) did not show statistically significant associations with ΔUACR. These findings highlight the critical role of baseline UACR and glycemic control (HbA1c) in influencing changes in albuminuria, and thus, should be taken into consideration.

### 3.4. Endothelial Dysfunction Biomarkers

**Figure 3:**
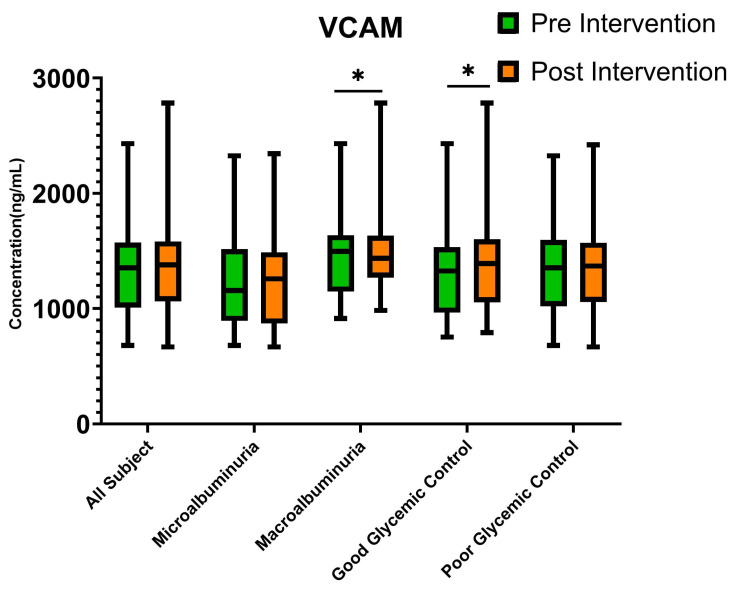
VCAM pre- and post intervention with sub-group analysis. * *p* < 0.05; hypothesis testing was conducted using the Wilcoxcon sign rank test. Microalbuminuria: UACR 30–300 mg/g at baseline; macroalbuminuria: UACR > 300 mg/g at baseline; good glycemic control: HbA1c ≤ 7.00; poor glycemic control: HbA1c > 7.00. VCAM: vascular cell adhesion molecule; total subjects n = 69, microalbuminuria n = 36, macroalbuminuria n = 36, good glycemic control n = 18, poor glycemic control n = 51.

To reveal the mechanism of action in reducing UACR, endothelial dysfunction biomarker levels were measured before and after intervention (Table 2). The levels of ICAM, VCAM, and VEGF did not significantly change after intervention (*p* > 0.05). However, based on analysis of change in UACR predictors after intervention, baseline UACR and Hb1c levels were identified as the most significant predictors. To analyze whether endothelial dysfunction plays role in how glycemic control and baseline UACR predict response to treatment, sub-group analysis of the endothelial dysfunction biomarker was conducted based on those categories. No significant differences were found in ICAM and VEGF based on any sub-group. However, subjects with good glycemic control showed a significant increase in VCAM after intervention (*p* = 0.048), with 1326.01 ng/mL (IQR 990.92–1522.84 ng/mL) at baseline and 1391.10 ng/mL (IQR 1077.64–1600.94 ng/mL) at four weeks after intervention (Figure 3). On the contrary, subjects with macroalbuminuria showed a significant decrease in VCAM (*p* = 0.046), with 1496.89 ng/mL (IQR 1163.47–1597.77 ng/mL) at baseline and 1436.97 ng/mL (1315.89–1616.83 ng/mL) at four weeks after intervention (Figure 3).

### 3.5. Correlation of Clinical Outcome Parameters with Endothelial Dysfunction Biomarkers

**Figure 4:**
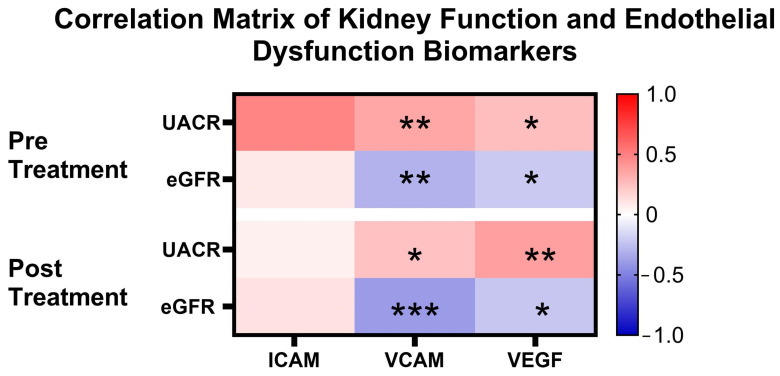
Correlation matrix of kidney function and endothelial dysfunction biomarkers. * *p* < 0.05; ** *p* < 0.01; *** *p* < 0.001; Correlational analysis was conducted using Spearman correlation. ICAM: intercellular adhesion molecule; VCAM: vascular cell adhesion molecule; VEGF: vasoendothelial growth factor.

In order to validate the relationship between kidney function and endothelial biomarker, correlation analysis was conducted. Figure 4 shows the results of the correlation between kidney function and endothelial dysfunction biomarkers before and after autologous dendritic cell immunotherapy. Before immunotherapy, UACR showed a significant correlation with VCAM (r = 0.346; *p* = 0.002) and VEGF (r = 0.252; *p* = 0.018), while there was no significant correlation with ICAM (r = 0.048; *p* = 0.349). After immunotherapy, the correlation between UACR and VCAM remained significant, though it decreased (r = 0.243; *p* = 0.022). The correlation between UACR and VEGF slightly increased (r = 0.376; *p* = 0.001). However, UACR still did not show a significant correlation with ICAM after immunotherapy (r = 0.057; *p* = 0.320).

Furthermore, before immunotherapy, eGFR showed a significant negative correlation with VCAM (r = −0.304; *p* = 0.006) and VEGF (r = −0.213; *p* = 0.039), while no significant correlation was found with ICAM (r = 0.087; *p* = 0.240). After immunotherapy, no meaningful changes in the correlations occurred. Although there was a slight increase in the negative correlation between eGFR and VCAM (r = −0.395; *p* = 0.000), this change remained significant. The negative correlation with VEGF also remained significant (r = −0.225; *p* = 0.031), indicating a consistent relationship between decreased eGFR and increased VEGF. The correlation between eGFR and ICAM remained non-significant post immunotherapy (r = 0.115; *p* = 0.173). These results indicate that endothelial dysfunction, as reflected by elevated levels of VCAM and VEGF, is linked to a decline in kidney function, as measured by eGFR and UACR, in DKD patients. The lack of significant changes in the strength of these correlations after immunotherapy suggests that the relationship remains largely unchanged.

## 4. Discussion

The majority of the subjects were in the elderly age group, which aligns with the high-risk population for chronic diseases such as hypertension and diabetes. This study population has a high risk for cardiovascular and kidney complications due to the high prevalence of hypertension, diabetes, obesity, and dyslipidemia. The combination of antihypertensive and antidiabetic treatments used reflects intensive efforts to control these risk factors. However, albuminuria and reduced eGFR indicates that subjects have already experienced significant kidney damage.

Subcutaneous injection was selected as the route of administration because the subcutaneous layer of the skin is rich in adipose tissue. Dendritic cells require migration to lymphatic organs to exert their full functionality. The initial dermal lymphatic vessels, which are open-ended, are located just above the adipose tissue layer in the skin [25]. Moreover, deposition into adipose tissue allows for a slower release of dendritic cells compared to the rapid flow characteristics of blood vessels like veins or arteries. This slow and sustained presence of DC is anticipated to increase the possibility of DC interaction with other immune cells.

This study showed a significant reduction in UACR after dendritic cell (DC) immunotherapy in DKD patients up to four weeks post intervention. Despite some weekly fluctuations, the overall median UACR remained lower compared to baseline. These results are comparable to standard therapies such as Telmisartan, Enalapril, GLP-1 RA, and SGLT2 inhibitors. These drugs can decrease UACR ranging from 17% to 65% after long-term use [26,27,28,29]. In this study, the analysis of the entire subject population revealed that DC immunotherapy reduced UACR by 25.41% ± 36.40% (mean ± standard deviation) at four weeks after intervention. Sub-group analysis revealed that the percentage of reduction between microalbuminuria and macroalbuminuria was not significantly different. This signifies that this treatment is effective in both subjects’ characteristics.

HbA1c and baseline UACR were identified as significant predictors of UACR change. The target HbA1c level for diabetes mellitus (DM) patients is <7%, making this cutoff value applicable for classifying glucose control among subjects [30]. Previous studies have revealed that higher HbA1c levels correlate with increased albumin excretion rates [31]. Chronic hyperglycemia contributes to glomerular damage through mechanisms such as advanced glycation end-product (AGE) accumulation, oxidative stress, and inflammation, all of which exacerbate albuminuria [32]. Consequently, HbA1c serves not only as a marker of metabolic status but also as a modifiable factor in therapeutic strategies aimed at mitigating renal damage. Baseline UACR, on the other hand, provides a direct measure of kidney damage at the initiation of treatment. Elevated UACR levels indicate significant glomerular injury, increased permeability, and heightened inflammatory activity. Its predictive value for subsequent UACR changes suggests that the severity of initial kidney damage profoundly influences the response to therapy. However, the negative impact of baseline UACR on UACR improvement observed in this study implies that after a certain degree of kidney damage, therapeutic interventions may become less effective due to the irreversible nature of the injury. This study did not limit the use of drugs that might affect albuminuria. Concomitant used of RAAS inhibitor, SGLT-2 inhibitor, or GLP-1RA did not contribute significantly to UACR change.

The analysis of creatinine levels pre- and post immunotherapy did not show any significant differences. Similarly, eGFR values before and after immunotherapy did not differ significantly. Comparisons with previous studies revealed varying results regarding changes in eGFR and creatinine after therapy in patients with kidney disease. Some studies report that in chronic kidney disease patients, especially those with high levels of albuminuria, improvements in eGFR after intervention are often minimal or not statistically significant, despite observable trends [26,27]. These studies suggest that reductions in albuminuria and improvements in eGFR may take longer to achieve statistical significance. Typically, the effects of therapy on kidney function are more apparent after a longer treatment period and with more aggressive combination therapies.

The improvement in UACR observed in this study suggests an improvement in kidney function after DC immunotherapy. This improvement is hypothesized to be mediated by enhanced endothelial function, as indicated by improvements in ICAM, VCAM, and VEGF levels. Previous research has shown increased expression of ICAM and VCAM in patients with kidney disorders, especially in diabetic kidney disease, supporting the hypothesis that these biomarkers play a role in the inflammatory process and endothelial damage [33,34]. Conversely, VEGF levels tend to be lower in DKD patients due to the loss of podocytes, the primary producers of VEGF in the kidney [35].

To confirm the relationship of endothelial biomarker with UACR, Spearman correlation analysis was conducted. Correlation analysis revealed that VCAM and VEGF have significant correlation with both UACR and eGFR. Our findings confirm previous studies which found that VCAM is a diagnostic biomarker of diabetic kidney disease progression that positively correlates with UACR and negatively correlates with eGFR [36]. VCAM is upregulated in response to inflammatory cytokines, which mediates immune cell infiltration and subsequent kidney damage [37]. On the other hand, VEGF is implicated in glomerular capillary hyperpermeability, contributing to diabetic albuminuria. In line with our findings, previous studies also found that a strong positive correlation exists between VEGF and UACR, indicating that higher VEGF levels are associated with increased albuminuria [37,38].

However, in this study, no significant changes were found in ICAM, VCAM, and VEGF biomarkers. While there were slight variations in median values, these changes were not substantial enough to be considered significant. This probably means that a decrease in UACR was not mediated by improvement of endothelial dysfunction. However, this should be interpreted with caution. There are other factors that should be considered. First, in comparison to previous studies analyzing changes in ICAM, VCAM, and VEGF, significant changes are often only seen after longer therapy durations or when combined with other interventions, such as improved glycemic control or additional pharmacological therapies directly affecting these biomarkers [39]. This suggests that the response to therapy may be cumulative and take longer to become evident in clinical trials. Second, previous studies have found that endothelial biomarkers like ICAM, VCAM, and VEGF tend to exhibit localized changes, which are not always reflected systemically [40]. In this study, biomarkers were measured from serum, representing systemic levels of ICAM, VCAM, and VEGF. However, it is possible that changes in these biomarkers were localized, making them undetectable in serum measurements.

Subgroup analysis revealed contrasting trends in VCAM changes: a decrease in VCAM levels among patients with macroalbuminuria and an increase in VCAM levels among those with good glycemic control. These findings suggest that the underlying mechanisms driving endothelial dysfunction and its resolution may differ depending on the baseline severity of kidney damage and glycemic control status. VCAM expression is upregulated in activated endothelial cells, facilitating the recruitment of inflammatory cells to sites of vascular damage [41]. In patients with macroalbuminuria, the observed decrease in VCAM may reflect a reduction in endothelial activation and inflammation following therapy, likely driven by the mitigation of severe vascular damage. This indicates that treatment may effectively reduce vascular inflammation in individuals with more advanced kidney damage. Conversely, the increase in VCAM levels in patients with good glycemic control could imply enhanced endothelial activity related to repair and regeneration rather than inflammation. VCAM enhances the migration and angiogenesis of bone marrow mesenchymal stem cells (BMSCs) which is important for repair and regeneration [42]. This group may exhibit an adaptive vascular response, where improved glycemic control supports endothelial recovery and function, leading to transient VCAM elevation.

This study has several limitations. First, this study evaluated changes in UACR, biomarkers and renal function within four weeks post immunotherapy. While significant reductions in UACR were observed, longer follow-up periods are required to assess sustained therapeutic effects, potential progression of kidney function, and delayed responses in biomarkers like ICAM, VCAM, and VEGF. Second, biomarkers like ICAM, VCAM, and VEGF were measured in serum, which reflects systemic levels. However, localized changes in these biomarkers within the kidney or specific vascular compartments may not be captured by systemic measurements, limiting the ability to fully assess endothelial dysfunction and repair mechanisms. Only a few endothelial biomarkers (ICAM, VCAM, and VEGF) were assessed. Other inflammatory and oxidative stress markers, which might provide a more comprehensive understanding of endothelial function and kidney health, were not included. Third, this study did not include a placebo or alternative therapy control group for direct comparison. While reductions in UACR were observed and compared with historical data on standard therapies, the absence of a control group limits the ability to attribute changes solely to dendritic cell immunotherapy. Multivariate analysis revealed that concomitant drug use did not significantly contribute to the UACR change. However, this does not exclude the possibility that these drugs might mask or even reduce the treatment effect of the intervention.

Future studies should address these limitations by incorporating longer follow-up periods, larger sample sizes, more comprehensive biomarker panels, and control groups. Such efforts will help validate the findings and provide deeper insights into the mechanisms and long-term efficacy of dendritic cell immunotherapy in diabetic kidney disease.

## 5. Conclusions

This study demonstrates that dendritic cell (DC) immunotherapy significantly reduces urinary albumin-to-creatinine ratio (UACR) in diabetic kidney disease (DKD) patients, suggesting its potential as an effective therapeutic approach. HbA1c and baseline UACR were identified as key predictors of UACR changes, highlighting the importance of glycemic control and baseline kidney damage in treatment outcomes. While no significant changes in endothelial biomarkers (ICAM, VCAM, and VEGF) were observed, the reduction in UACR indicates the involvement of other mechanisms, possibly localized endothelial repair or immune modulation. Subgroup analysis revealed contrasting VCAM trends, suggesting that endothelial responses vary based on glycemic control and the severity of kidney damage. These findings support the potential of DC immunotherapy for DKD, though further studies with longer follow-up and larger sample sizes are needed to validate and expand on these results.

## Figures and Tables

**Table 1:** Creatinine and estimated glomerular filtration rate levels.

Parameter	Pre Intervention	Post Intervention	*p*-Value
Creatinine (mg/dL)	1.04 (0.82–1.62)	1.12 (0.78–1.66)	0.467
eGFR (mL/min/1.73 m^2^)	60.35 (38.78–86.31)	56.26 (37.59–90.07)	0.478

Hypothesis testing was conducted using the Wilcoxon sign rank for all parameters. Data were presented as median (IQR).

**Table 2:** Endothelial dysfunction biomarker (ICAM, VCAM, VEGF) levels.

Parameter	Pre Intervention	Post Intervention	*p*-Value
ICAM (ng/mL)	314.50 ± 93.12	315.12 ± 78.98	0.905
VCAM (ng/mL)	1354.25 (1015.90–1567.03)	1380.65 (1067.92–1572.03)	0.827
VEGF (pg/mL)	504.44 (294.54–790.72)	494.57 (319.39–775.10)	0.664

Hypothesis testing was conducted using the Wilcoxon sign test for VCAM and VEGF, and paired t-test for ICAM. VCAM and VEGF were presented as median (IQR). ICAM was presented as mean ± SD.

## Data Availability

The raw data supporting the conclusions of this article will be made available by the authors on request.

## Supplementary Materials

The following supporting information can be downloaded at: https://www.mdpi.com/article/10.3390/cimb47010031/s1, Table S1: Subject Characteristics.
