# Supplementary material for: The Effect of Autologous Dendritic Cell Immunotherapy on Kidney Function and Endothelial Dysfunction of Patients with Diabetic Kidney Disease (DKD): An Open Label Clinical Trial
Source: Curr Issues Mol Biol. 2025 Jan 6;47(1):31. doi: 10.3390/cimb47010031 (PMC11764056; doi:10.3390/cimb47010031)
Supplement: Supplementary file 1 [file cimb-47-00031-s001.zip › cimb-3362519-Supplementary.pdf]

Supplementary Table S1. Subject Charateristics

**Characteristics**	**All Subjects (n=69)**	**Microalbuminuria (n=36)**	**Macroalbuminuria (n=33)**	**p-value**
**Age**				
Mean (range) year	62,04 (39-83)	63 (44-83)	61 (39-82)	0,434
**Sex**				
Male	30 (43,5%)	14 (46,7%)	14 (42,4%)	0,422
Wanita	39 (56,5%)	22 (56,4%)	19 (57,6%)	0,422
**Race**				
Jawa	29 (42%)	17 (47,2%)	12 (36,4%)	0,260
Tionghoa	9 (13%)	5 (13,9%)	4 (12,1%)	
Betawi	8 (12%)	3 (8,3%)	5 (15,2%)	
Batak	8 (12%)	5 (5,6%)	3 (9,1%)	
Sunda	5 (7%)	2 (5,6%)	3 (9,1%)	
Minang	4 (6%)	3 (8,3%)	1 (3%)	
Others	6 (9%)	1 (2,8%)	5 (15,2%)	
**Comorbidity**				
Hypertension	65 (94,2%)	35 (97,2%)	31 (93,9%)	0,266
Heart Disease	29 (42%)	23 (63,9%)	21 (63,6%)	0,950
Stroke	6 (8,7%)	15 (41,7%)	14 (42,4%)	0,377
Neuropathy	38 (55,1%)	11 (30,5%)	13 (39,3%)	0,572
Retinopathy	7 (10,1%)	5 (13,9%)	2 (6%)	0,286
Osteoarthritis	20 (29,0%)	3 (8,3%)	4 (12,1%)	0,449
Other	27 (39,1%)	11(30,5%)	16 (48,5%)	0,594
**Anti-hipertensive Drugs**				
ARB	49 (71%)	24 (66,7%)	25 (75,8%)	0,409
ACE-i	44 (63,8%)	21 (58,3%)	23(69,7%)	0,460
Beta Blocker	23 (33,3%)	15 (41,7%)	7 (21,2%)	0,091
CCB	6 (8,7%)	4 (11,1%)	2 (6%)	0,330
Alpha blocker	4 (5,8%)	1 (2,8%)	3 (9%)	0,610
Diuretik	3 (4,3%)	2 (5,6%)	1 (3%)	0,266
**Anti-diabetic Drugs**				
Insulin	44 (63,8%)	22 (61,1%)	22 (66,7%)	0,634
Sulphonylurea	29 (42%)	15 (41,7%)	14 (42,4%)	0,950
Biguanide	22 (31,9%)	12 (33,3%)	10 (30,3%)	0,789
SGLT2-inhibitor	15 (21,7%)	6 (16,7%)	9 (27,3%)	0,290
DPP4-inhibitor	13 (18,8%)	7 (19,4%)	6 (18,2%)	0,894
α-glucosidase-i	12 (17,4%)	9 (25%)	3 (9,1%)	0,084
Thiazolidinedione	1 (1,4%)	1 (2,8%)	0	0,338
**BMI**				
<18.5 kg/m^2(underweight)	2 (2,9%)	2 (100%)	0	0,64
18.5-24.9 kg/m^2 (normal weight)	21 (30,4%)	15 (71.4%)	6 (28.6%)	
≥25.0 kg/m^2 (overweight)	35 (50,7%)	15 (42.9%)	20 (57.1%)	
≥30.0 kg/m^2 (obesity)	11 (15,9%)	4 (36.4%)	7 (63.6%)	
**Total Cholesterol**				
<200 mg/dl	40 (58%)	24 (66,7%)	16 (48,5%)	0,126
>200 mg/dl	29 (42%)	12 (33,3%)	17 (51,5%)	
**Trigliseride** (*interquartile range)* mg/dL	145 (100-187)	140.55 mg/dL	187.36 mg/dL	0,26
**LDL** (*interquartile range)* mg/dL	115(93-158)	113.69 mg/dL	134.61 mg/dL	0,041*
**HDL** (*interquartile range)* mg/dL	47(41-53)	47.81 mg/dL	46.76 mg/dL	0,704
**eGFR Classification**				
>90 mL/min/1.73m²	16 (23,2%)	9 (25%)	6 (18,2%)	0,340

60-89 mL/min/1.73m^2	21 (30,4%)	12 (33,3%)	9 (27,3%)	
45-59 mL/min/1.73m^2	9 (13%)	3 (8,3%)	8 (24,2%)	
30-44 mL/min/1.73m^2	23 (33,4%)	12 (33,3%)	10 (30,3%)	
P-values were determined by comparing Microalbuminuria and Macroalbuminuria groups. The P-value for "Age" was calculated using an independent t-test. P-values for "Triglycerides, LDL, and HDL" were calculated using the Mann-Whitney U test. P-values for "Sex, Race, Comorbidities, Anti-Hypertensive Drugs, Anti-Diabetic Drugs, BMI, Total Cholesterol, and eGFR Classification" were calculated using either the Chi-square test or Fisher's Exact test, as appropriate.				
